# A surrogate weighted mean ensemble method to reduce the uncertainty at a regional scale for the calculation of potential evapotranspiration

**DOI:** 10.1038/s41598-020-57466-0

**Published:** 2020-01-21

**Authors:** Byoung Hyun Yoo, Junhwan Kim, Byun-Woo Lee, Gerrit Hoogenboom, Kwang Soo Kim

**Affiliations:** 1 Department of Plant Science, Seoul National University, Seoul, 08826 Korea; 2 Crop Production & Physiology Division, National Institute of Crop Science, Rural Development Administration, Wanju-gun, Jeollabuk-do, 55365 Korea; 3 Department of Agricultural and Biological Engineering, University of Florida, Gainesville, 32611 USA; 4 Institute for Sustainable Food Systems, University of Florida, Gainesville, 32611 USA; 5 Research Institute of Agriculture and Life Sciences, Seoul National University, Seoul, 08826 Korea

**Keywords:** Climate and Earth system modelling, Hydrology

## Abstract

We propose a weighted ensemble approach using a surrogate variable. As a case study, the degree of agreement (DOA) statistics for potential evapotranspiration (PET) was determined to compare the ordinary arithmetic mean ensemble (OAME) method and the surrogate weighted mean ensemble (SWME) method for three domains. Solar radiation was used as the surrogate variable to determine the weight values for the ensemble members. Singular vector decomposition with truncation values was used to select five ensemble members for the SWME method. The SWME method tended to have greater DOA statistics for PET than the OAME method with all available models. The distribution of PET values for the SWME method also had greater DOA statistics than that for the OAME method over relatively large spatial extent by month. These results suggest that the SWME method based on the weight value derived from the surrogate variable is suitable for exploiting both diversity and elitism to minimize the uncertainty of PET ensemble data. These findings could contribute to a better design of climate change adaptation options by improving confidence of PET projection data for the assessment of climate change impact on natural and agricultural ecosystems using the SWME method.

## Introduction

A regional impact assessment of climate change can help with the development of adaptation strategies to climate change^1,2^. These impact assessment studies are often accompanied by projection of an ecological variable using future climate change scenario data as inputs to ecological models and agronomic models^3–5^. However, the compounded effect of uncertainty inherent in the climate input data to the models can affect the projected results^6^. A multi-model ensemble approach has been used to minimize the uncertainty of the projection data^7,8^. For example, Asseng *et al*.^9^ reported that an ensemble approach had the potential to reduce the uncertainty of wheat yield projections using a large number of crop simulation models.

Ensemble approaches based on the average of ensemble members can be classified into two groups according to weight values. The ordinary arithmetic mean ensemble (OAME) method assigns an equal weight to each member to determine an ensemble value of multiple model outputs^10^. The OAME method is the simplest approach to obtain an ensemble projection once outputs from multiple models become available. Alternatively, the weighted arithmetic mean ensemble (WAME) method can be used to reduce the uncertainty of projection data^11^. In the WAME method, a weight value is often determined to be reciprocal to the magnitude of the uncertainty for an ensemble member. The WAME method has been applied to gridded estimations of meteorological variables such as temperature^12^ and precipitation^13^. Different approaches, including the Reliability Ensemble Average (REA)^11^ and the Taylor Skill Score (TSS)^14^, have been used to determine the weight value for each ensemble member.

The WAME method has the potential to minimize the uncertainty of climate change projections^15^. Nevertheless, it has only been applied in a relatively small number of studies especially for the spatial assessment of climate change impact on natural and agricultural ecosystems. It is rather challenging to determine the weight value of an ensemble member for ecological variables because these variables often have little availability of observation data over a region. For example, measurements of evapotranspiration (ET) are available mostly for a specific area where a flux tower or lysimeter is installed, but not for a region. The lack of observed data, therefore, would limit the application of the WAME method for gridded outputs of ET^16^.

An alternative approach can be used to take advantage of the WAME method without observed data for a variable of interest. In this approach, a surrogate variable is selected among variables that would have great availability of observed data. The variable of interest would be highly sensitive to the surrogate variable. In the surrogate-weighted mean ensemble (SWME) scheme, a weight value is derived from the surrogate variable to represent the relative importance of an ensemble member. For example, a surrogate variable can be used for ensemble projection of potential evapotranspiration (PET), which is one of key variables to assess the impact of climate change on water resources and crop yield^17^. PET is usually calculated using a function of solar radiation, temperature, and other weather variables^18^. Bois *et al*.^19^ reported that PET tended to have greater sensitivity to solar radiation than other forcing variables. Thus, solar radiation can be used as the surrogate variable to reduce the uncertainty of PET projection data.

The objectives of this study were to develop and evaluate an alternative ensemble approach using a surrogate variable, which could aid a reliable projection of environmental variables with a relatively small number of ensemble members. Application of the SWME method could increase the confidence of projection data even for the variables for which the observation data are limited. In particular, a small number of ensemble members can be chosen from a large pool of ensemble members available for a region. The surrogate variable could allow for assessment of interdependency among ensemble members without observation data for the variable of interest. This then could improve climate change impact assessments of natural and agricultural ecosystems.

## Materials and Methods

### Reference and ensemble PET

The value of PET was determined using global reanalysis data and regional downscaled data. AgMERRA data, which are the global reanalysis data developed for comparison and improvement of agricultural simulation models^20^ were used to prepare the reference data for PET (PET_AgMERRA_). AgMERRA data were obtained from the Goddard Institute for Space Studies website (https://data.giss.nasa.gov/impacts/agmipcf/agmerra/, accessed at 07 November 2019). Daily data for four variables, including surface air temperature, surface downwelling solar radiation, specific humidity, and wind speed, were used as inputs to the FAO56 equation^18,21^.

**Figure 1:**
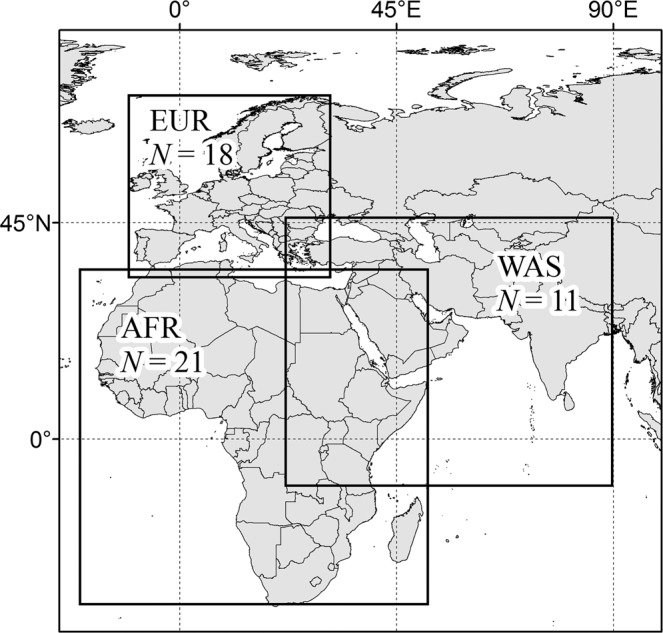
Extent of domains used to calculate potential evapotranspiration (PET). These domains include Africa (AFR), Europe (EUR) and West Asia (WAS). *N* indicates the number of all available model for the domain.

The reference data of PET were compared with ensembles of PET for three domains (Fig. 1). The Coordinated Regional Climate Downscaling Experiment (CORDEX) data, which consist of the outputs of multiple regional climate models (RCM), were used to create the PET ensembles. In the CORDEX program, regional climate data were created in 13 domains^10,22,23^ downscaling the outputs of global circulation models (GCM) (Supplementary Fig. S1). It would be preferable to quantify the degree of agreement between reference and ensemble data using a large set of reginal climate data. In the present study, there were three domains where more than 10 sets of regional climate data were available. The CORDEX data in these domains were obtained from Earth System Grid Federation website (https://esgf-data.dkrz.de/, Accessed at 07 November 2019).

The CORDEX data were resampled to have comparable spatial properties to the global data. The spatial resolution of AgMERRA and CORDEX data is 0.25° and 0.5°, respectively. These data also have different projection. The CORDEX Data Support Library suggested by Yoo and Kim^21^ was used to reproject these CORDEX data to the latitude-longitude grid using WGS84 datum, which is comparable to the AgMERRA data. The extent of CORDEX^24^ data was adjusted to have a rectangular shape (Fig. 1).

Ensemble data sets were grouped by time periods. The weight value for the surrogate variable were determined for the period of 1981–1990, which was denoted by the baseline period. Data from 1991–2000 were used for the evaluation of PET ensemble. Another evaluation dataset was prepared using AgMERRA dataset for the period of 2001–2005.

### Ensemble methods

The ensemble of PET was created using the ordinary arithmetic mean (PET_OAME_) and surrogate-weighted arithmetic mean (PET_SWME_) of ensemble members, respectively. In a domain, the daily data of an RCM included in the CORDEX were used to calculate the ensemble member of PET (PET_RCM_). The PET_OAME_ was created as follows

1 $${{\rm{PET}}}_{{\rm{OAME}}}=\sum {{\rm{PET}}}_{{\rm{RCM}}}\cdot 1/N,$$

where *N* is the number of RCMs or ensemble members in the given domain. The values of PET_SWME_ were calculated as follows:

2 $${{\rm{PET}}}_{{\rm{SWME}}}=\sum {{\rm{PET}}}_{{\rm{RCM}}}\cdot {w}_{RCM},$$

where *w*_RCM_ indicates the weight value derived from the surrogate variable for an ensemble member RCM. The value of *w*_RCM_ was determined comparing the values of surrogate variable for the AgMERRA data and the CORDEX data as follows:

3 $${w}_{RCM}={{\rm{TSS}}}_{{\rm{RCM}}}/\sum {{\rm{TSS}}}_{{\rm{RCM}}},$$

where TSS_RCM_ represents the TSS of the surrogate variable for RCM. TSS was selected as a weighting scheme because it would require relatively small number of ensemble members in a short term periods^25^. Average value of solar radiation for the baseline period in a domain was used to determine the value of TSS_RCM_ as follows^14^:

4 $${{\rm{TSS}}}_{{\rm{RCM}}}=4(1+{R}_{{\rm{RCM}}})/{({\hat{s}}_{{\rm{RCM}}}+1/{\hat{s}}_{{\rm{RCM}}})}^{2}(1+{{\rm{R}}}_{0}),$$

where *R*_RCM_ and ${\hat{s}}_{{\rm{RCM}}}$ represent the correlation coefficient and the ratio of standard deviation between PET_AgMERRA_ and PET_RCM_ in a domain. R_0_, which is the maximum attainable correlation coefficient, was set to one as Suh *et al*.^15^ suggested. The weight value for an ensemble member was determined using the skill score of solar radiation for the baseline period of 1981–1990. The same weight values were applied to the periods of 1991–2000 and 2001–2005 to calculate ensembles of PET for the SWME method.

### Assessment of interdependency among ensemble members

It would be preferable to use a small number of ensemble members unless the uncertainty of the ensemble would increase. For example, Knutti *et al*.^26^ suggested that a subset of ensemble members can be chosen to have comparable confidence to that of ensemble using all the available ensemble members. Sanderson *et al*.^27^ suggested a simple approach to select a specific number *n* of ensemble members assessing interdependency between these members. In particular, they reported that the scores of quality and uniqueness for ensemble members could result in improvement of confidence of an ensemble.

The Uniqueness Score (US) for the surrogate variable was determined using the distance between ensemble members as Sanderson *et al*.^27^ suggested (Supplementary Fig. S2). The singular value decomposition with truncation to *t*-mode was used to calculate the distance. In the present study, TSS of the surrogate variable for ensemble members was determined to replace the root mean square error used in their study.

Independence quality score (IQS) was calculated summing the product of US and TSS for a given number *n* of ensemble members (Supplementary Fig. S2). To remove the ensemble member that caused a low value of IQS, an iterative process was performed to determine IQS for *n-*1 ensemble members. The subset of ensemble members that had the maximum IQS was chosen to go through the next iteration until the desired number *n* of ensemble members remained. The values of TSS were used as the weight values for each ensemble member to obtain PET_SWME-*n*-*t*_ for a given *t*-mode.

A set of ensemble members was chosen among multiple sets of the members derived from a series of truncation values. The truncation values ranged from 5 to 12^27^. As a result, eight sets of ensemble members were identified for each *n*. In the present study, the coefficient of variation (CV) of the skill scores for ensemble members was used for the selection criteria. For a given number of ensemble members, the CV value of the skill score for the surrogate variable was calculated as follows:

5 $${\rm{CV}}={s}_{{\rm{TSS}}}/{m}_{{\rm{TSS}}},$$

where *s*_TSS_ and *m*_TSS_ indicate the standard deviation and average of TSS_RCM_ values for ensemble members, respectively. The set of *n* ensemble members was used to calculate the values of PET.

Diversity among ensemble members were taken into account to select the set of ensemble members for calculation of PET. The CV values of the TSS among *n* ensemble members were determined for each set of ensemble members for the truncation value (Supplementary Fig. S2). The truncation value *t*_max-cv_ with which the maximum CV values, CV_max_, were obtained from the given sets of *n* ensemble members was identified. The set of ensemble members corresponding to the value of *t*_max-cv_ was chosen for the calculation of PET, which was denoted by PET_SWME-*n*_.

Information entropy *I* of the TSS values was calculated to choose an alternative set of ensemble members. The *I* value was determined as follows:

6 $$I=-\sum {{\rm{tss}}}_{i}\cdot {{\rm{logtss}}}_{i},$$

where tss_*i*_ is the normalized value of the TSS for given ensemble member *i*. The set of ensemble members that had the maximum *I* value, *I*_max_, were used to prepare another ensemble of PET, PET_SWME-*n*-entropy_. The truncation value for the chosen set of ensemble members was denoted by *t*_max*-I*_.

### Degree of agreement statistics

All available ensemble members in a domain were used for the OAME method to prepare an ensemble dataset, PET_OWME-all_. The values of PET_SWME-*n*_ and PET_OWME-all_ were compared to examine if the confidence of the ensemble data can be maintained using a considerably small number of ensemble members. The degree of agreement (DOA) statistics were determined to compare two types of PET ensembles. The Concordance Correlation Coefficient (CCC) was used to evaluate the overall DOA between averages of reference and ensemble data over a region for a given period. The CCC has been used to evaluate both accuracy and precision of estimates^28^. Daily data for PET ensemble were averaged during the given period by cell for the domain of interest. The CCC value for the domain was calculated as follows^29^:

7 $${\rm{CCC}}=2\cdot R\cdot {s}_{{\rm{AgMERRA}}}\cdot {s}_{{\rm{Ensemble}}}/[{s}_{{\rm{AgMERRA}}}^{2}+{s}_{{\rm{Ensemble}}}^{2}+({m}_{{\rm{AgMERRA}}}-{m}_{{\rm{Ensemble}}})],$$

where *R* represents the correlation coefficient between the maps of PET_AgMERRA_ and PET_Ensemble_. *m*_Data_ and *s*_Data_ indicate the average and the standard deviation of a given PET dataset *Data* among cells, respectively. The values of CCC were determined for the periods of 1981–1990, 1991–2000 and 2001–2005, which were denoted by CCC_1980s_, CCC_1990s_, and CCC_2000s_, respectively. The CCC values of the PET_SWME-*n*_ were compared with those of the PET_OAME-all_.

The Kolmogorov-Smirnov test statistic *d* was determined by month to examine the similarity between the distribution of reference and ensemble data for PET_SWME-*n*_ and PET_OAME-all_. The value of *d* was calculated for each cell using daily values of PET in a month over a given period, e.g., 1981–1990. A smaller value for *d* indicates that an ensemble method has a similar distribution of PET to the reference data at a greater degree. The number of cells where the *d* value was less for one method compared to another method was determined to examine difference in the spatial extent of uncertainty between the ensemble methods.

## Results

### Comparison between selection indices for ensemble members

The variability of a skill score for the surrogate variable was useful for selection of a truncation value for assessment of interdependency among ensemble members (Supplementary Fig. S3). The value of Concordance Correlation Coefficient (CCC) for PET was relatively high for truncation values that resulted in greater value of the coefficient of variation (CV) for the TSS for solar radiation. In particular, the CCC value was the greatest for the ensemble members chosen to have the maximum variability among the pool of ensemble members.

**Figure 2:**
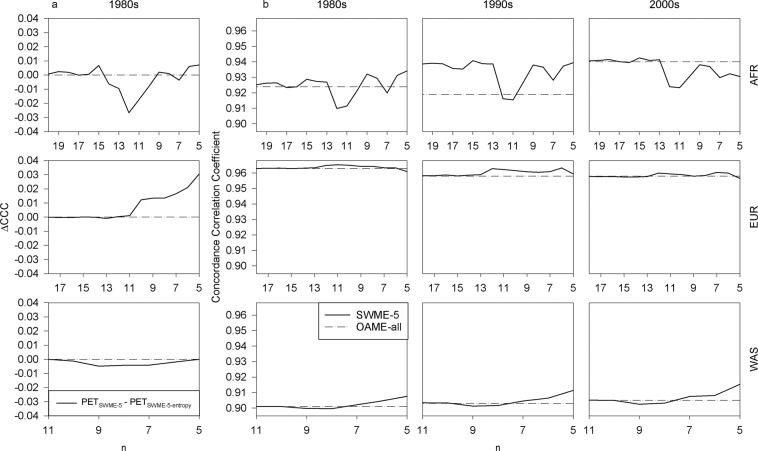
Comparison of Concordance Correlation Coefficient (CCC) values for potential evapotranspiration (PET) ensemble using a given number of ensemble members. The difference of CCC for PET using coefficient of variation and information entropy for the skill score as selection criteria for ensemble members. (**a**) The CCC values for surrogate-weighted mean ensemble (SWME-*n*) methods using a given number *n* of ensemble members. (**b**) All the available ensemble members in a given domain were used for the ordinary arithmetic mean ensemble (OAME-all). AFR, EUR and WAS indicate Africa, Europe, and West Asia, respectively.

It was of greater advantage to select ensemble members using the CV of the skill score for the surrogate variable than the information entropy (Fig. 2a). PET_SWME-*n*_ and PET_SWME-*n-*entropy_, which are the PET ensemble obtained from *n* ensemble members chosen to maximize the CV value and the information entropy, respectively, had relatively similar values of degree of agreement (DOA) statistics. The difference between the CCC values for PET_SWME-*n*_ and PET_SWME-*n*-entropy_, ΔCCC, mostly ranged from −0.005 to 0.005. However, the CCC values for PET_SWME-*n*_ was relatively high when a small number of ensemble members were used. On the other hand, PET_SWME-*n*-entropy_ tended to have higher values of CCC than PET_SWME-*n*_ for a large value of *n*. For example, PET_SWME-*n*_ had high values of CCC for Europe and Africa domains when five ensemble members were used (Supplementary Fig. S4). PET_SWME-*n*-entropy_ usually had relatively higher values of CCC for Africa domain than PET_SWME-*n*_ using 10–14 members were used (Fig. 2a). In the West Asia domain, the difference between CCC of PET_SWME-*n*_ and PET_SWME-*n*-entropy_ was relatively small using any number of members (Fig. 2a).

### Degree of agreement statistics of PET ensembles

The number of ensemble members did not necessarily affect the CCC values for the SWME method (Fig. 2b). For example, the SWME method had larger values of the CCC_1980s_ than the OAME method did when five ensemble members were used for Africa and West Asia domains. On the other hand, the CCC values for the SWME method were relatively low even when a large number of ensemble members, e.g., more than 10, were used (Fig. 2b). The SWME method had smaller values of the CCC_1980s_ and the CCC_1990s_ than the OAME method when the former was applied to 12 ensemble members for the Africa domain.

It has been reported that the use of five ensemble members could improve the confidence of ensemble data^30,31^. We also found that the DOA statistics for the averages of PET tended to be greater for the SWME method than the OAME method when the five ensemble members were selected for the SWME method (Table 1). For example, the CCC_1980s_ values of *PET*_*OAME-all*_ using 21 ensemble members were considerably high (0.924) for the Africa domain. However, the CCC_1980s_ values for PET_SWME-5_ were even greater (0.934) using only five ensemble members.

**Table 1:** Concordance Correlation Coefficient (CCC) of the PET ensemble for Africa (AFR), Europe (EUR), and West Asia (WAS) domains during the periods of 1981–1990, 1991–2000, and 2001–2005, respectively.

DOMAIN	1981–1990		1991–2000		2001–2005	
	OAME-all	SWME-5	OAME-all	SWME-5	OAME-all	SWME-5
AFR	0.924	0.934	0.919	0.939	0.94	0.931
EUR	0.963	0.961	0.958	0.959	0.958	0.957
WAS	0.901	0.908	0.903	0.911	0.905	0.915

The ordinary arithmetic mean ensemble (OAME-all) and the surrogate-weighted mean ensemble (SWME-5) methods were applied to all the available ensemble members and five ensemble members, respectively.

When PET_SWME-5_ had a greater value of CCC than PET_OAME-all_, the magnitude of difference between these ensemble data were relatively large (Table 1). For example, the Africa domain had relatively large differences between PET_SWME-5_ and PET_OAME-all_ during the periods of 1981–1990 and 1991–2000. On the other hand, PET_SWME-5_ and PET_OAME-all_ for the Europe domain had similar CCC values during these periods when the CCC values for PET_SWME-5_ was relatively smaller than those for PET_OAME-all_.

### Spatial distribution of bias

**Figure 3:**
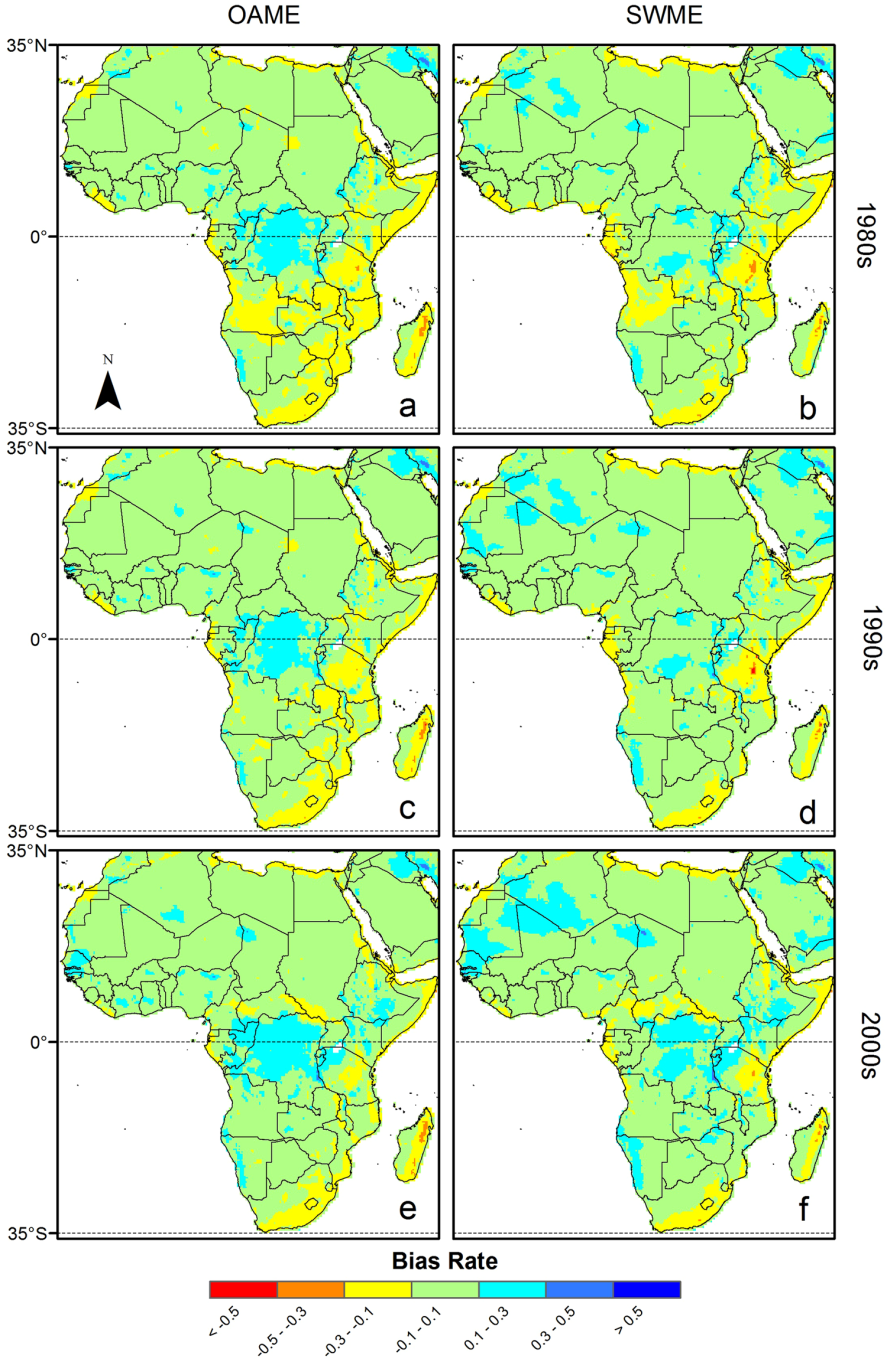
Spatial distribution of biases for potential evapotranspiration (PET) using two ensemble methods in the Africa domain. The time periods denoted by 1980s, 1990s, and 2000s ranged from (**a**,**b**) 1981–1990, (**c**,**d**) 1991–2000 and (**e**,**f**) 2001–2005, respectively. The negative value of bias rate represents under-estimation bias. The PET values were obtained from (**a**,**c**,**e**) the OAME method and (**b**,**d**,**f**) the SWME method using 21 and five ensemble members, respectively.

**Figure 4:**
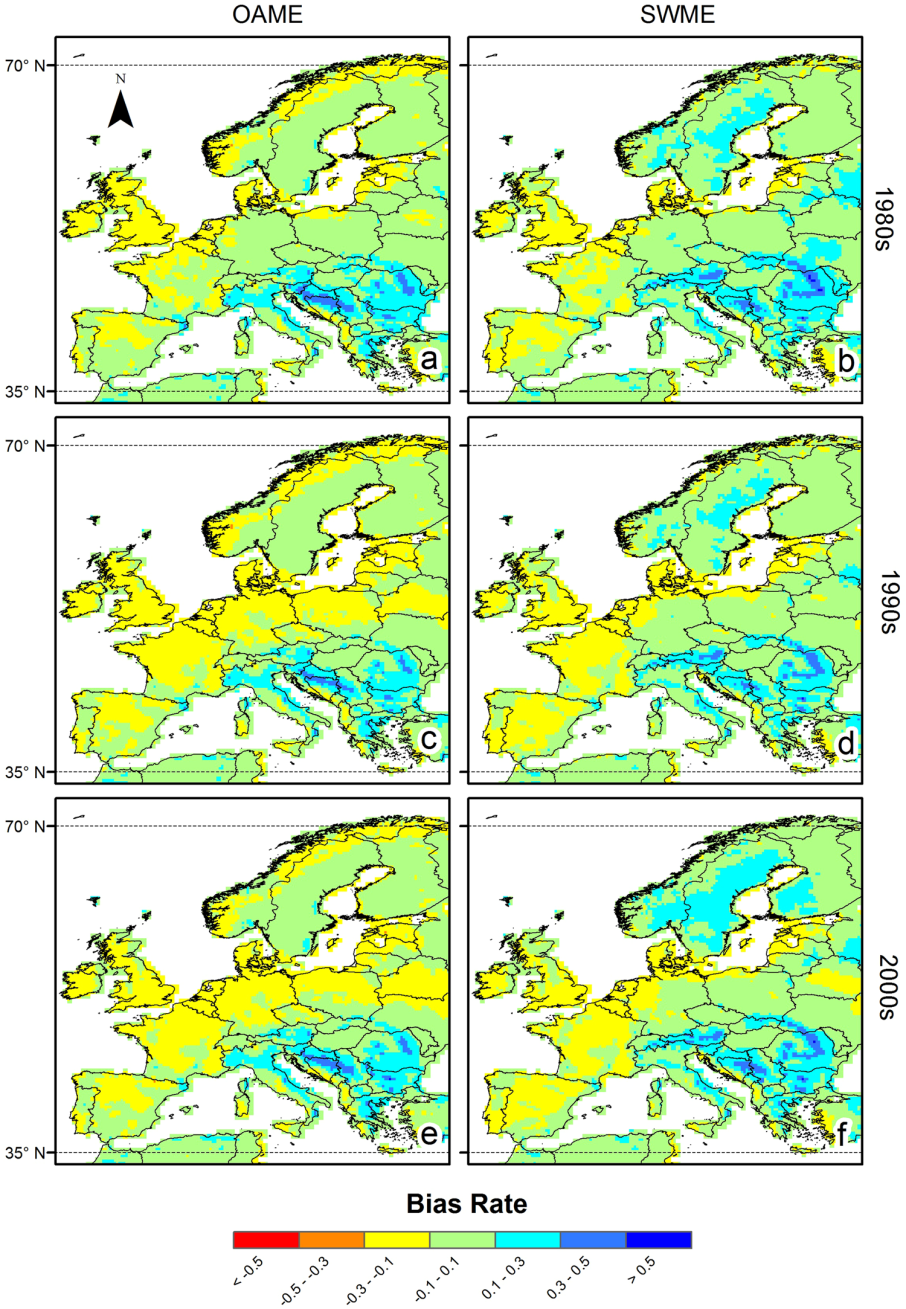
Spatial distribution of biases for potential evapotranspiration (PET) using two ensemble methods in the Europe domain. The time periods denoted by 1980s, 1990s, and 2000s ranged from (**a**,**b**) 1981–1990, (**c**,**d**) 1991–2000 and (**e**,**f**) 2001–2005, respectively. The negative value of bias rate indicates under-estimation bias. The PET values were obtained from (**a**,**c**,**e**) the OAME method and (**b**,**d**,**f**) the SWME method using 18 and five ensemble members, respectively.

**Figure 5:**
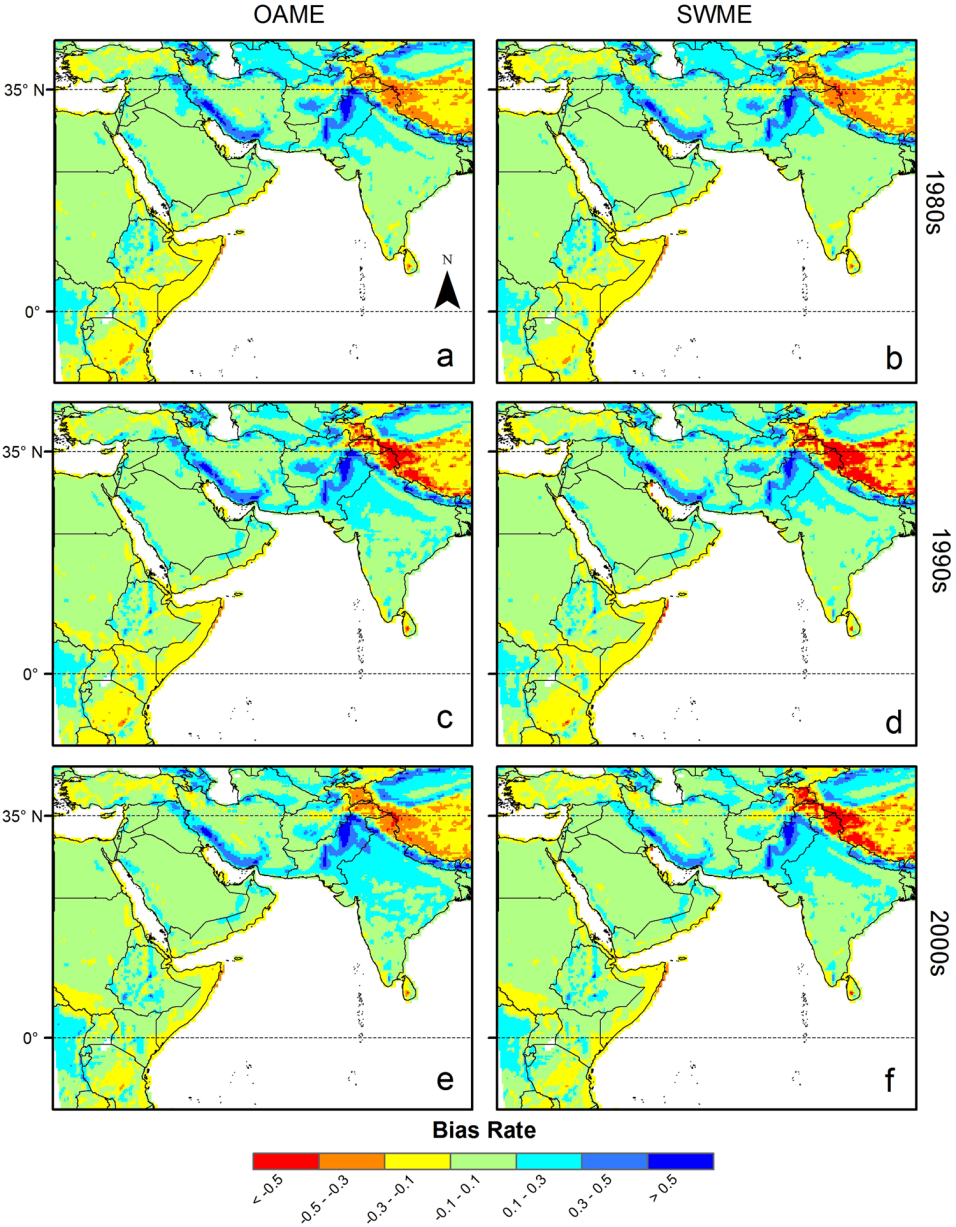
Spatial distribution of biases for potential evapotranspiration (PET) using two ensemble methods in the West Asia domain. The time periods denoted by 1980s, 1990s, and 2000s ranged from (**a**,**b**) 1981–1990, (**c**,**d**) 1991–2000 and (**e**,**f**) 2001–2005, respectively. The negative value of bias rate indicates under-estimation bias. The PET values were obtained from (**a**,**c**,**e**) the OAME method and (**b**,**d**,**f**) the SWME method using 11 and five ensemble members, respectively.

The spatial distribution of the bias for the PET ensemble was similar between ensemble methods for the given domains (Figs. 3–5). In the Africa domain, for example, there were regions where relatively similar magnitude of bias occurred over the periods for each ensemble method. The positive bias occurred in larger areas than the negative bias for both SWME and OAME methods. Nevertheless, the SWME method had larger areas where the bias between reference and ensemble PET data was small, e.g., within 10%, except for the period from 2001–2005. Similar results were obtained for the other domains. For instance, PET_SWME-5_ had smaller bias in the western part of Europe, e.g., Germany and Poland, than PET_OAME-all_. In contrast, both PET_SWME-5_ and PET_OAME-all_ had similar positive bias in Eastern Europe.

**Figure 6:**
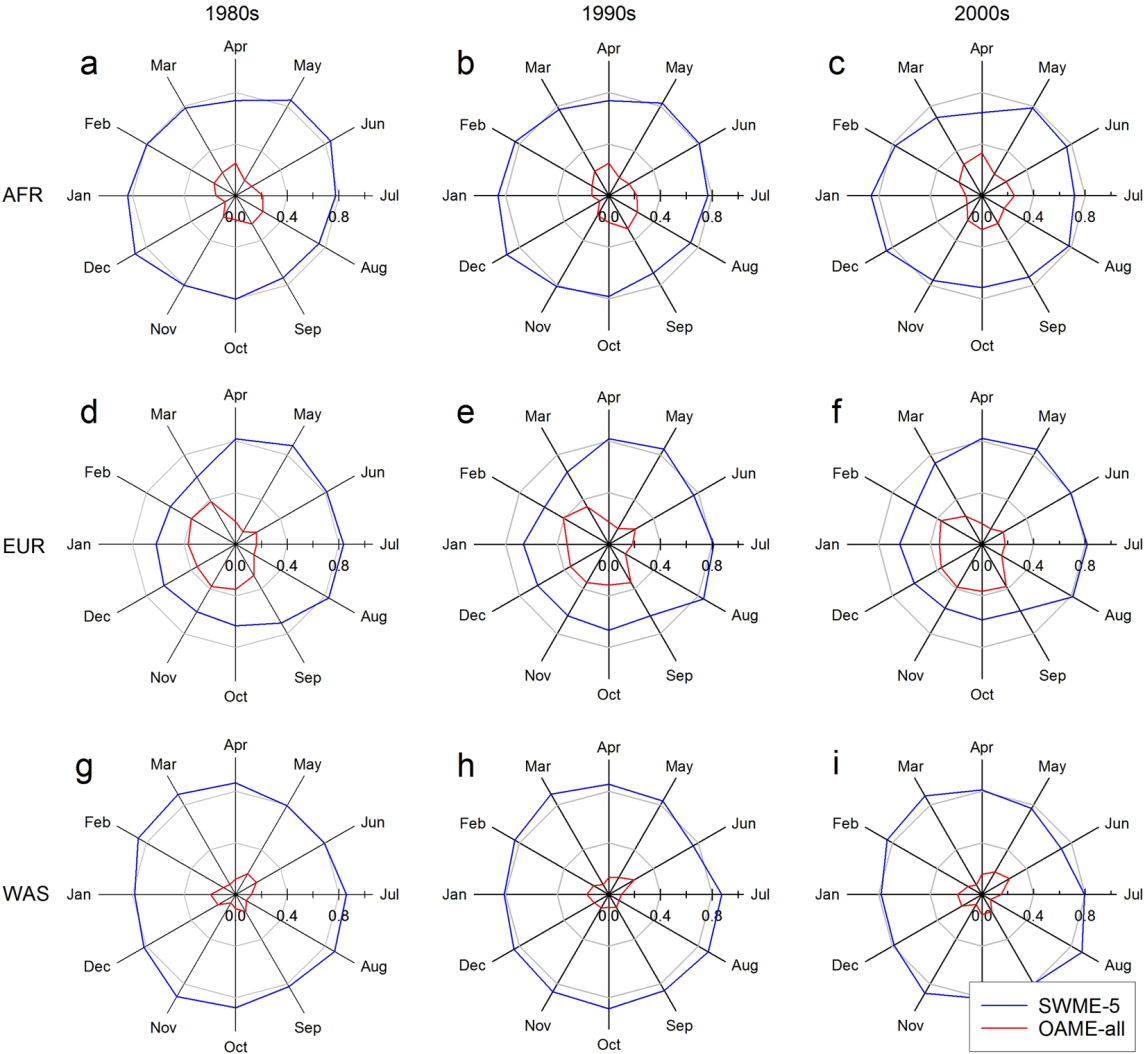
The fraction of areas where the Kolmogorov-Smirnov test statistic *d* of potential evapotranspiration (PET) ensemble was smaller for a given ensemble method than the other method. Ordinary arithmetic mean ensemble (OAME-all) and surrogate-weighted mean ensemble (SWME-5) methods were used to obtain PET ensemble. The values for *d* were determined for (**a**–**c**) Africa (AFR), (**d**–**f**) Europe (EUR) and (**g**–**i**) West Asia (WAS) by month during the periods of (**a**,**d**,**g**) 1981–1990, (**b**,**e**,**h**) 1991–2000, and (**c**,**f**,**i**) 2001–2005, respectively. The radial plots indicate the fraction of cells where the value of *d* for daily PET projection data for a given month was smaller for one ensemble method compared to another method. Five ensemble members were used for the SWME-5 method whereas all available ensemble members for the domains were used for the OAME-all method.

The SWME method resulted in the similar distribution of daily values of PET to the reference data at a greater degree over a larger area compared with the OAME method (Fig. 6). For example, the PET_SWME-5_ had a relatively small value of Kolmogorov-Smirnov statistic *d* for a greater number of cells than the PET_OAME-all_ during the period of 1981–1990 for the Africa domain (Supplementary Fig. S5). Such results were sustained for the periods of 1991–2000 and 2001–2005 (Supplementary Figs. S6, 7). The extent where PET_SWME-5_ had smaller values of *d* than PET_OAME-all_ was relatively similar between months for Africa and West Asia domains, which was about >80% of the region. In the Europe domain, on the other hand, there was seasonal variation in the extent where the *d* values for PET_SWME-5_ were smaller than those for PET_OAME-all_. Still, the *d* values for the PET _SWME-5_ were relatively small for the greater number of cells compared with PET_OAME-all_ for the given domain.

## Discussion

### Diversity and elitism of the ensemble members

Laumanns *et al*.^32^ suggested that the combination of diversity and elitism would be effective for optimization. The ensemble method is based on the assumption that the uncertainty would be canceled out between ensemble members^33^. Ensemble members with the higher variability would have the greater statistical averaging effect, which has been reported in multi-model ensemble studies^34^. The diversity of ensemble members can be quantified using a score for independence among ensemble members. The degree of elitism or the relative importance of an ensemble member has been taken into account using the skill score for the variable of interest. Still, this approach would require the observation data for the variable of interest to assess diversity and elitism of ensemble members.

Our results illustrate that a surrogate variable would allow for the evaluation of diversity and elitism of ensemble members without the observed data for the variable of interest. Independence and skill scores, which would be corresponding to the scores for diversity and elitism, respectively, have been assessed to improve the confidence of ensemble using the observation data^35^. In the present study, diversity among ensemble members was assessed only for the surrogate variable. The surrogate variable was also used to quantify the elitism of individual ensemble members without using observation data for PET.

### Advantages of the SWME method

Weigel *et al*.^36^ suggested that the OAME method would be preferable to the WAME method for ensemble studies due to the limitations in determining the optimum weight values for the ensemble members. For example, the observation data would be needed to determine weight values for ensemble members. The weight values determined for a period could also have the over-confidence for another period. Still, the OAME method often requires a large number of ensemble members to reduce uncertainty^37^. Kharin *et al*.^38^ reported that the skill of an ensemble increased consistently when more than six members were used for the OAME method. However, in practice, it would be challenging to use a large number of ensemble members especially for ecological models and agronomic models because it would require prohibitively large computing resources^39^.

Our findings suggest that the SWME method could help reduce the uncertainty of the spatial assessment of climate change impact using a model to predict an ecological variable with a small number of ensemble members. The overall accuracy of a variable for the SWME method was at least comparable to that of OAME method. The former had greater spatial extent where the distribution of ensemble data for the ecological variable was closer to that of the reference data than the latter. This indicates that the SWME method had improved the confidence of the spatial assessment of the variable, which could aid the design of climate change adaptation options over a region.

### Limitations of the SWME method

The sensitivity of ecological and agricultural models can be affected by multiple variables including non-climate variables. The use of a single surrogate variable may have a limited impact on increasing confidence of the ensemble data. For example, Folberth *et al*.^40^ suggested that a crop growth model had greater sensitivity to soil characteristics than climatic variables under certain conditions, e.g., no fertilizer or irrigation. The sensitivity of a model to a climate variable may differ by region^41^, which suggests that identification of surrogate variable by region would be needed. Application of multiple surrogate variables including both non-climate variable and climatic variables would be needed to improve the confidence of ensemble data using the SWME method. For example, a machine learning approach can be applied to explore an integrated impact of different surrogate variables using the outputs of an ecological model^42^. We also found that the degree of agreement statistics could differ by season for the SWME method (Supplementary Fig. S8). Thus, the SWME method could be further improved by using weight values at a shorter temporal scale (Supplementary Fig. S8). This suggests that the SWME method would provide an opportunity to reduce the uncertainty in the projection of an ecological variable, which merits further application to diverse climate change projection studies in natural and agricultural ecosystems.

## Supplementary information

Supplementary Information.

## Data Availability

Ensembled average potential evapotranspiration data are available at “https://figshare.com/s/3ca00543071fec6db00e”
