# Supplementary material for: A surrogate weighted mean ensemble method to reduce the uncertainty at a regional scale for the calculation of potential evapotranspiration
Source: Sci Rep. 2020 Jan 21;10:870. doi: 10.1038/s41598-020-57466-0 (PMC6972760; doi:10.1038/s41598-020-57466-0)
Supplement: Supplementary file 1 — Supplementary Information. [file 41598_2020_57466_MOESM1_ESM.docx]

**A surrogate weighted mean ensemble method to reduce the uncertainty at a regional scale for the calculation of potential evapotranspiration**

Byoung Hyun Yoo^1^, Junhwan Kim^2^, Byun-Woo Lee^1^, Gerrit Hoogenboom^3,4^, and Kwang Soo Kim^1,5*^

Supplementary Information


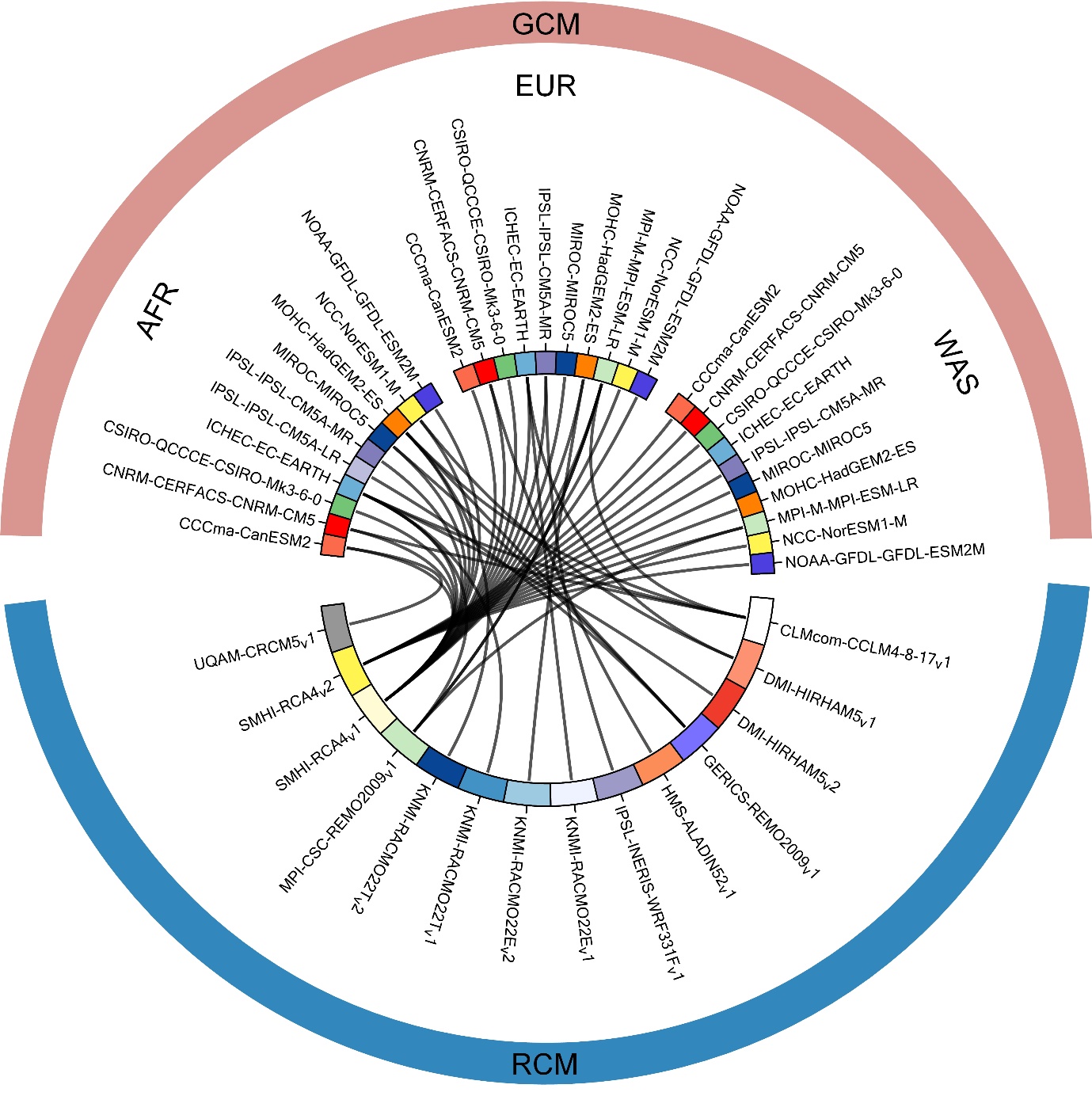


Fig. S1. Relationship between Regional Climate Models (RCM) and Global Circulation Models (GCM) to create climate data in the CORDEX domains. A line indicates a pair of RCM and GCM to perform the dynamical downscaling of climate data. For example, two RCMs were used for downscaling the outputs of 10 GCMs in the West Asia (WAS) domain. In both Africa (AFR) and Europe (EUR) domains, outputs of 10 GCMs were downscaled using eight RCMs.


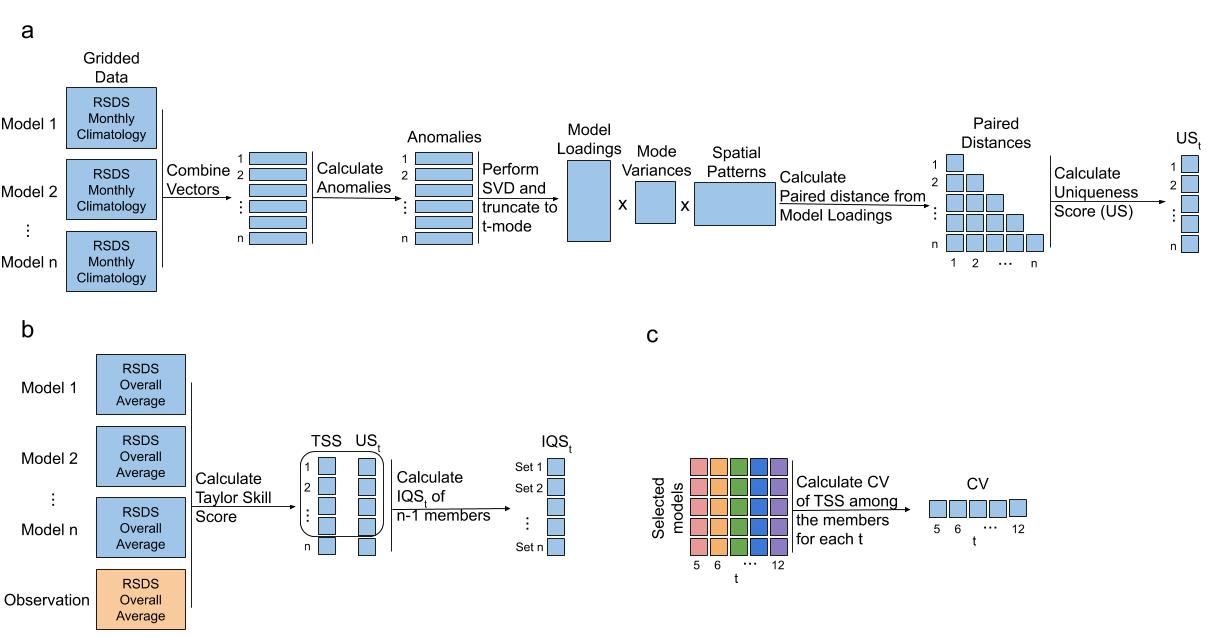


Fig. S2. The schematic view of the process to assess interdependency among ensemble members. (a) The singular value decomposition was performed using monthly climate data for the surrogate variable to determine uniqueness score (US) as suggested by Sanderson *et al.* ^1^. (b) Taylor Skill Score (TSS) was determined and multiplied to US to compute Independence quality score (IQS) for each ensemble with n-1 members. (c) The coefficient of variation (CV) of the TSS for each set of ensemble members were calculated for the corresponding truncation value.

**~~
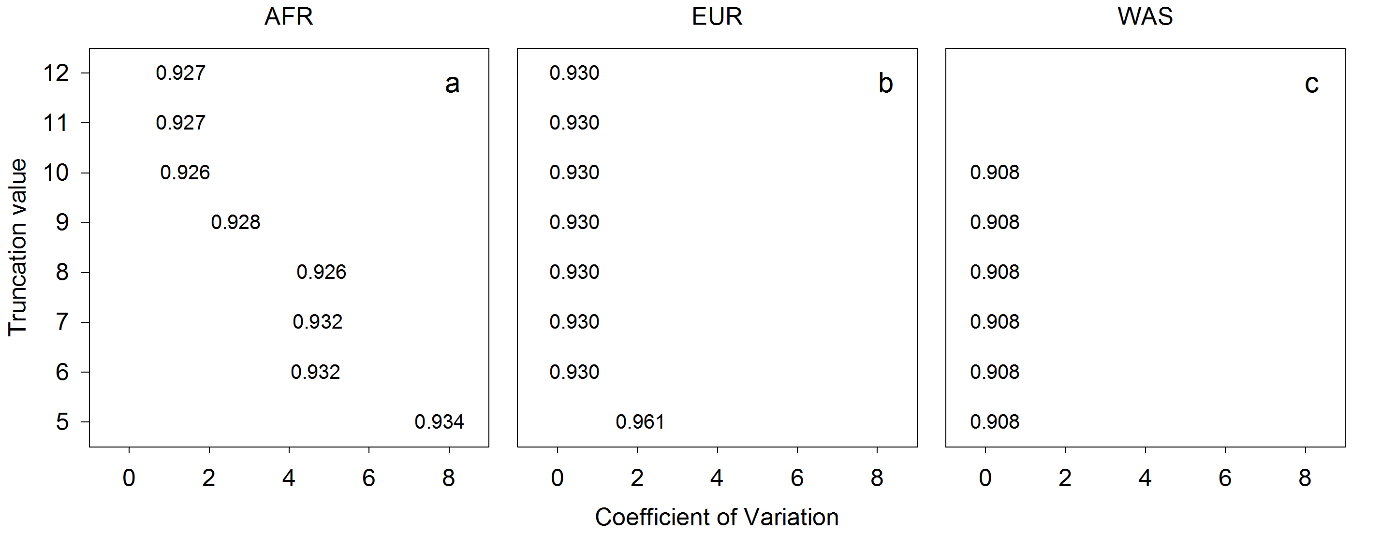
~~**

Fig. S3. The Concordance Correlation Coefficient (CCC) values of potential evapotranspiration (PET) for ensemble members chosen by truncation value. The variability among ensemble members were assessed using the coefficient of variation (CV) for the Taylor Skill Score of the surrogate variables. The surrogate weighted mean ensemble (SWME) method was applied to five ensemble members during the period of 1981-1990. The eight sets of five ensemble members were identified for the truncation values. The CCC values were relatively high for ensemble members with large variability. The values inside the plot indicate the magnitude of the CCC values. (a) AFR, (b) EUR and (c) WAS represent the domains of Africa, Europe and West Asia, respectively.


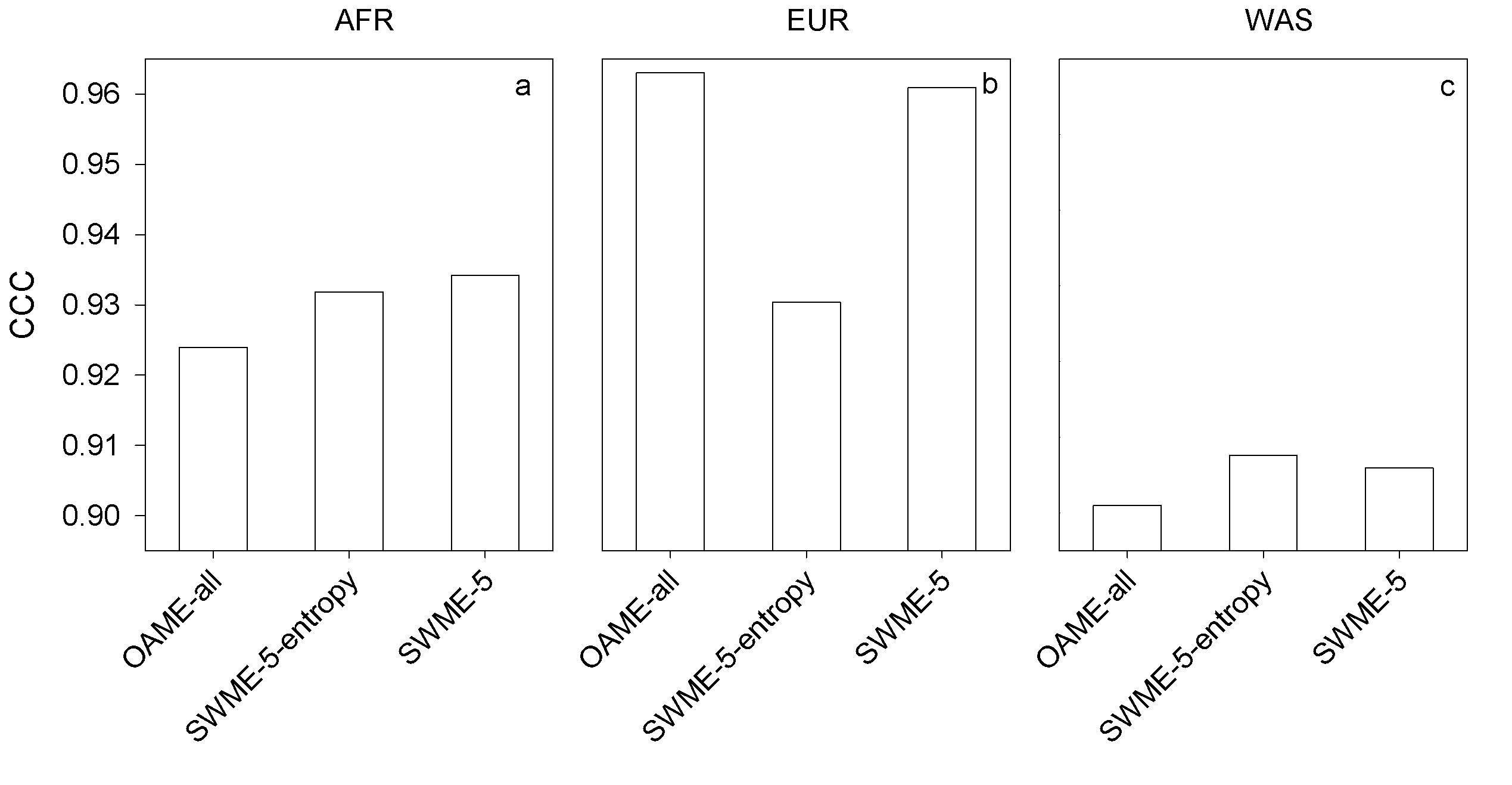


Fig. S4. The Concordance Correlation Coefficient (CCC) of potential evapotranspiration (PET) for different ensemble methods during the baseline period (1981-1990) by domain. The ordinary arithmetic mean ensemble (OAME-all) method was used for all available ensemble members for the given domain. The surrogate weighted mean ensemble (SWME) method was applied to five ensemble members chosen from different approaches. The selection criteria for the ensemble members were based on the maximum value of coefficient of variation (SWME-5) and information entropy (SWME-5-entropy) for the Taylor Skill Score of the surrogate variable, respectively. (a) AFR, (b) EUR and (c) WAS represent domains of Africa, Europe and West Asia, respectively.


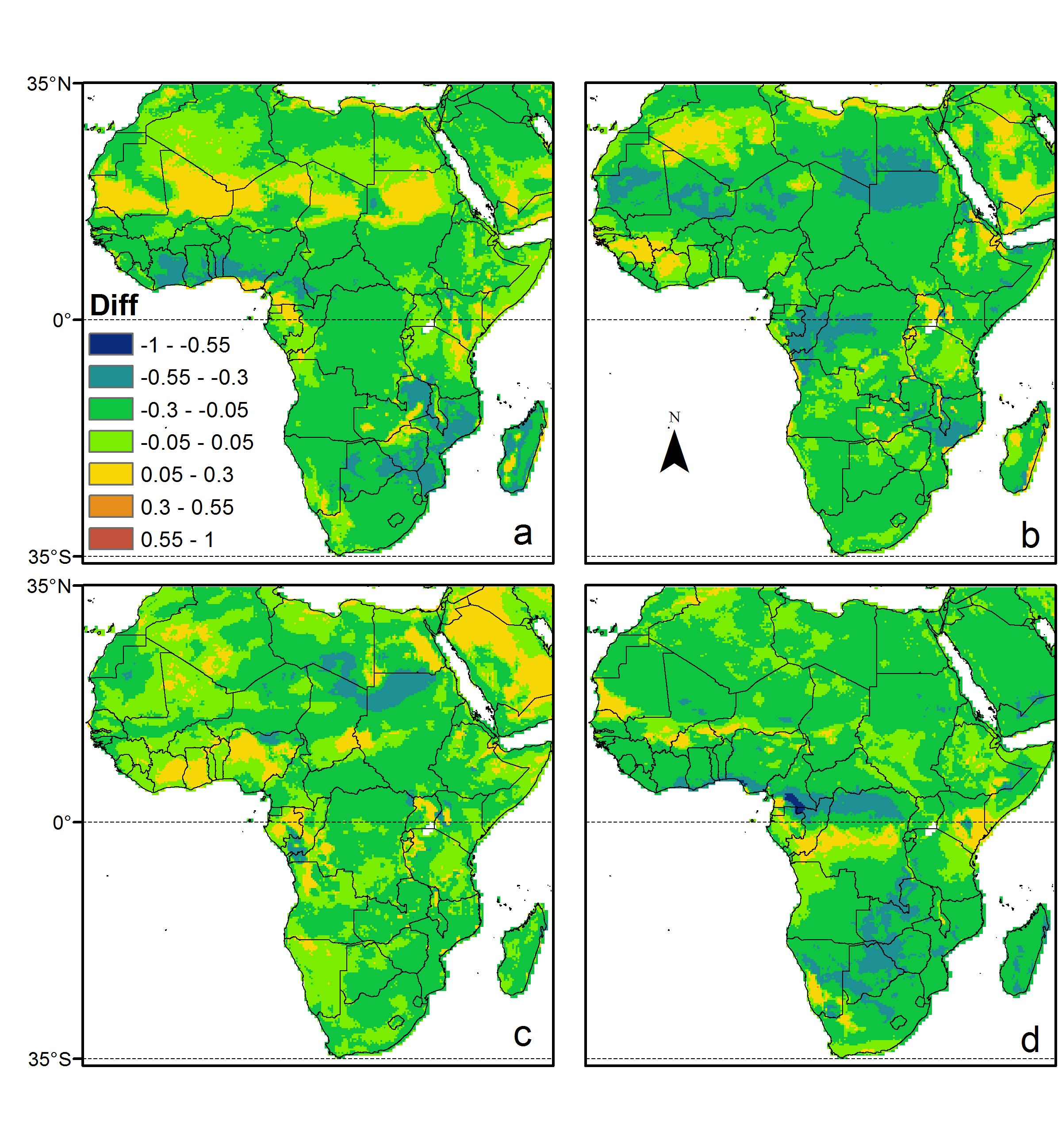


Fig. S5. Spatial difference (Diff) between the values of the Kolmogorov-Smirnov test statistic *d* for potential evapotranspiration ensemble using two methods in a given month during the period of 1981-1990. The SWME method was used for five ensemble members with the maximum variability whereas the OAME method was used for all available ensemble members (21 members). The daily values of PET were compiled to calculate the *d* value for each cell for (a) March, (b) June, (c) September, and (d) December. The negative value indicates that the *d* value for the SWME method was smaller than that for the OAME method.


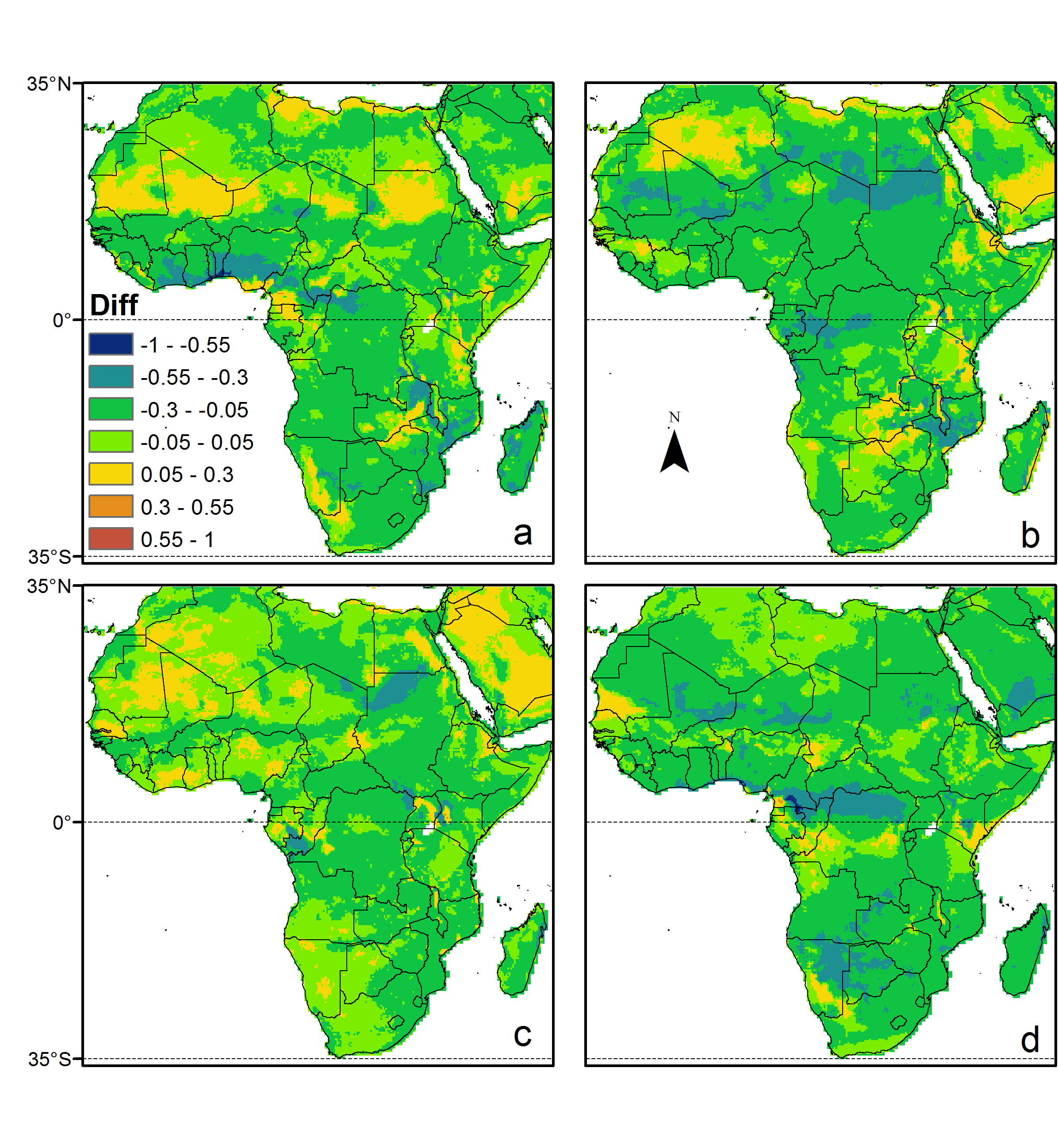


Fig. S6. Spatial difference (Diff) between the values of the Kolmogorov-Smirnov test statistic *d* for potential evapotranspiration ensemble using two methods in a given month during the period of 1991-2000. The SWME method was used for five ensemble members with the maximum variability whereas the OAME method was used for all available ensemble members (21 members). The daily values of PET were compiled to calculate the *d* value for each cell for (a) March, (b) June, (c) September, and (d) December. The negative value indicates that the *d* value for the SWME method was smaller than that for the OAME method.


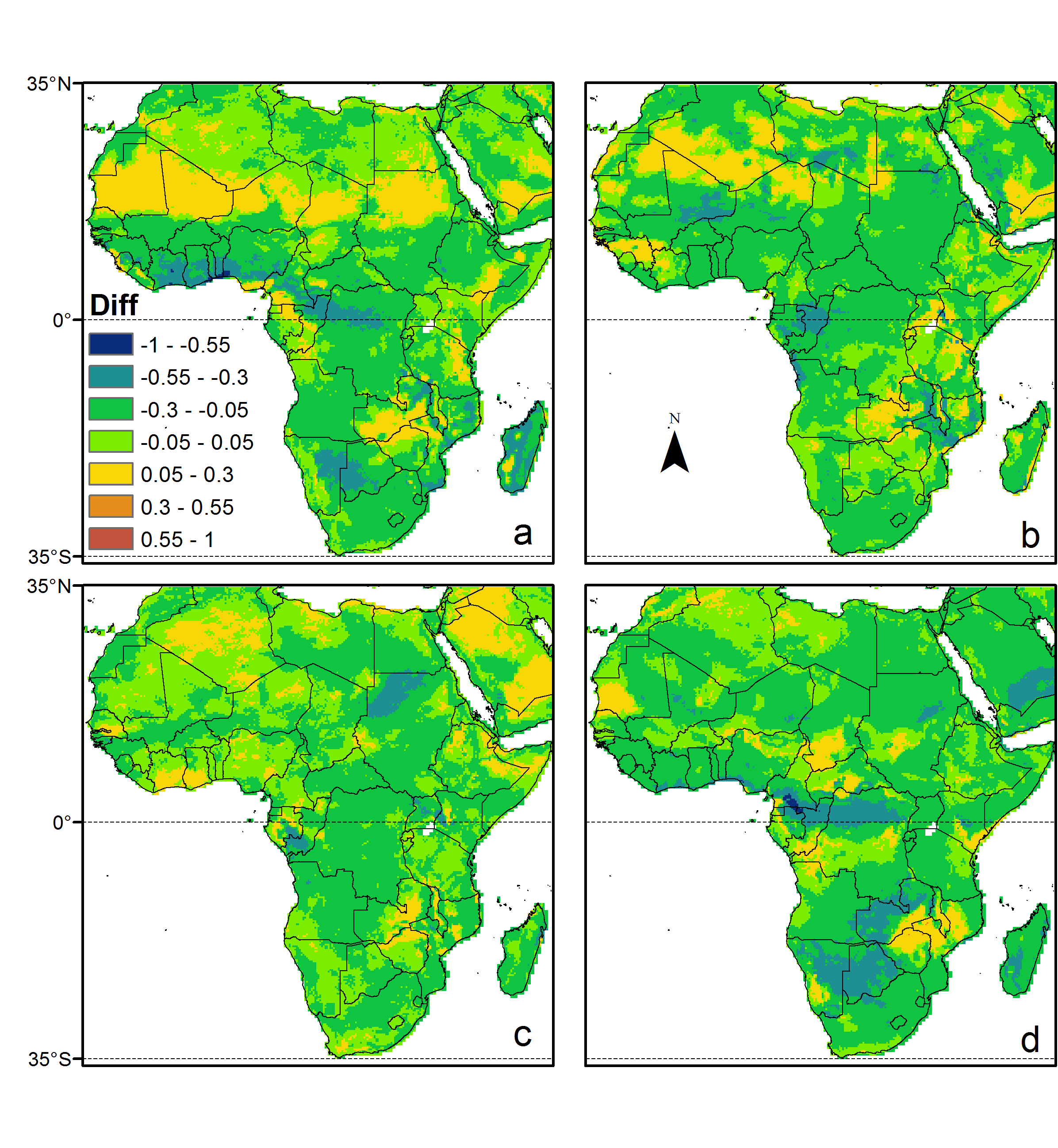


Fig. S7. Spatial difference (Diff) between the values of the Kolmogorov-Smirnov test statistic *d* for potential evapotranspiration ensemble using two methods in a given month during the period of 2001-2005. The SWME method was used for five ensemble members with the maximum variability whereas the OAME method was used for all available ensemble members (21 members). The daily values of PET were compiled to calculate the *d* value for each cell for (a) March, (b) June, (c) September, and (d) December. The negative value indicates that the *d* value for the SWME method was smaller than that for the OAME method.


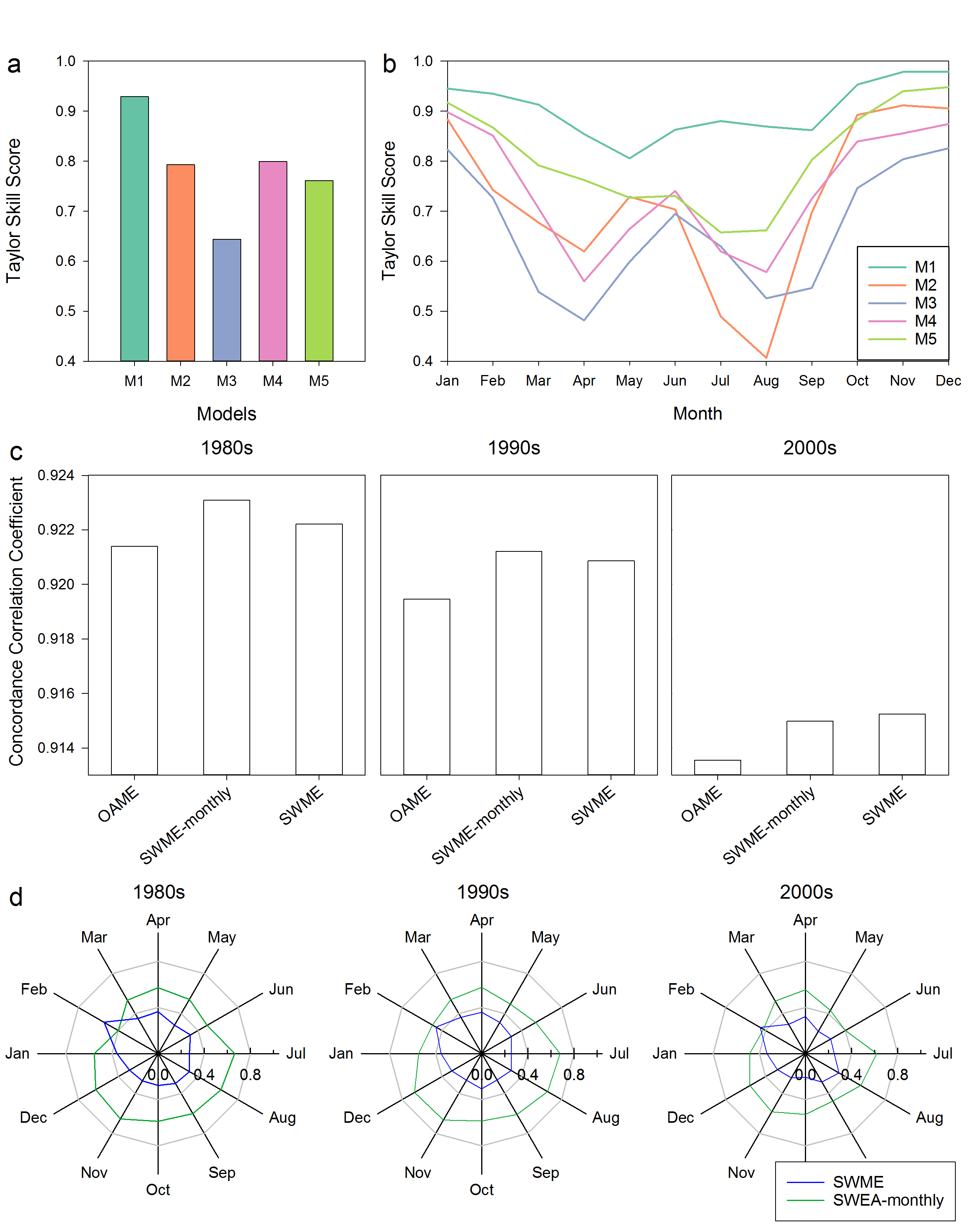


Fig. S8. Application of annual and monthly weights to the surrogate variables for calculation of potential evapotranspiration (PET) for the East Asia (EAS) domain (Supplementary Table S1). The Taylor Skill Score for each ensemble member was determined for (a) annual and (b) monthly averages of surrogate variables. These skill score values were used to determine weight values for ensemble members. (c) The Concordance Correlation Coefficient (CCC) of PET was determined during the periods of 1981-1990, 1991-2000 and 2001-2005 by the weight value set. The ordinary arithmetic mean ensemble (OAME) method, surrogate weighted mean ensemble (SWME), and monthly surrogate weighted mean ensemble (SWME-monthly) were used for all available (five) ensemble members for East Asia domain. (d) The ensemble data of PET using the SWME-monthly method had greater areas with relatively small values of the Kolmogorov-Smirnov test statistic *d* than those using SWME method.

Table S1. List of CORDEX East Asia data^a^ used in the present study

| GCM | INSTITUTE | RCM |
| --- | --- | --- |
| HadGEM2-AO | NIRM | HadGEM3-RA_v1 |
| HadGEM2-AO | YSU | YSU-RSM_v3 |
| HadGEM2-AO | SNU | SNU-WRF_v3 |
| HadGEM2-AO | KNU | RegCM_v4 |
| ICHEC-EC-EARTH | DMI | DMI-HIRHAM5_v1 |

^a^ http://cordex-ea.climate.go.kr accessed at 04 March 2018

**REFERENCES**

1. Sanderson, B. M., Knutti, R. & Caldwell, P. A Representative Democracy to Reduce Interdependency in a Multimodel Ensemble. *J. Clim.* **28**, 5171-5194, doi:10.1175/jcli-d-14-00362.1 (2015).
